# Hyper-Uricemia and Gouty Access in the Adult Population of the Southeast of Gabon: Biochemical Aspects

**DOI:** 10.3390/diseases6010019

**Published:** 2018-03-08

**Authors:** Guy Roger Ndong Atome, Rick-Leonid Ngoua Meye Misso, Cédric Sima Obiang, Richard Onanga, Dieudonné Nkogue Mba

**Affiliations:** 1 Laboratory of Research in Biochemistry (LAREBIO), University of Sciences and Technology of Masuku, Franceville P.O. Box 943, Gabon; ngouamartel@gmail.com (R.-L.N.M.M.); cedricsima@gmail.com (C.S.O.); 2 International Medical Research Center of Franceville (CIRMF), Franceville P.O. Box 769, Gabon; onangar@yahoo.com (R.O.); dnkoghe@hotmail.com (D.N.M.); 3 Ministry of Public Health of Gabon, Libreville BP 50, Gabon

**Keywords:** uric acid, hyperuricemia, gout access, metabolism, risk factor

## Abstract

Gout is caused by a chronic hyperuricemia whose complications are not currently well evaluated in Africa. The aim of this study was to determine the prevalence and risk factors of hyperuricemia and gout in 85 patients recruited. A total of 26 cases of hyperuricemia, i.e., 30.6% of the study population, with 12 cases of gout and seven cases of gouty access. In this population, hyperuricemia was proportional to age (*p*-value < 10^−4,^ OR = 2.6), but it was more prevalent in men, 23.5% versus 7.1% for women (*p*-value = 0.0047). In addition, none of these women showed signs of a gouty affection. Consumption of alcohol (OR = 13) and nucleoprotein-rich foods, obesity (BMI 30 kg/m^2^; OR = 6), family history of gout (OR = 6.8), as well as diseases such as high blood pressure (associated with taking diuretics; OR = 1.7), renal insufficiency (OR = 4.4) and diabetes (*p* < 0.049) were the main factors of the diseases associated with gout and hyperuricemia in this population. The biochemical role of these factors may increase and/or decrease the processes of synthesis and/or elimination of uric acid by acting on metabolites involved in the regulation of urate production.

## 1. Introduction

Unlike other endemic or epidemic diseases in the world, gout is one of the pathologies that act silently. It has long been marginalized to the detriment of life-threatening disease [1]. This underestimation, particularly in the sub-Saharan zone, is mainly due to the expertise limits of practitioners in its diagnosis [2]. From the 1980s, the expansion of more sophisticated diagnostic means in the West and in Africa demonstrated the real existence of rheumatic diseases including gout [3,4].

Gout is described as localized inflammatory arthritis caused by chronic hyperuricemia. The burden of this disease and its complications remain significant and rapidly increasing [5], but the rate of hyperuricemia and gout prevalence vary widely by region and especially by gender and age. Indeed, several studies have shown changes in the prevalence of hyperuricemia, particularly in Taiwan, where a case-control study conducted in 2012 on 2145 patients showed that more women than men are hyperuricaemic, respectively 43% and 35%, aged between 40 and 59 years [6]. In the West, however, the prevalence of hyperuricemia is higher in men than in women, i.e., between 15% and 20% for men and between 2% and 10% for women [7]. In addition, gouty access is the consequence of several risk factors such as alcoholism and overeating, and is generally associated with several various diseases such as hypertension, myocardial insufficiency, and kidney stones.

This work has demonstrated the reality of rheumatic diseases, such as gout, which is becoming a priority, especially in Africa. This is why Gabon is certainly not exempt from this disease, which could potentially be a handicap for the working population. In addition, currently no study data specific to hyperuricemia and gout made in Gabon is available. To get an idea of gouty profile, a pilot study was conducted to better report the risk factors in the adult population of the province of Haut-Ogooué in the southeast, and establish causal correlations involving the biochemical aspects of these factors.

## 2. Materials and Methods

### 2.1. Ethical Clearance

This study was reviewed and approved by the National Committee on Ethics for Research (CNER) of Gabon. Participants were informed that participation is completely voluntary, and written consent was obtained from each participant before being subjected to the questionnaire and after discussing the objective with the participants. No names were recorded on the questionnaires. Adequate training of data collectors took place to ensure protection of confidentiality, and all questionnaires were kept safe.

### 2.2. Area and Study Population

A prospective and transversal pilot study was conducted within the Medical Research and Analysis Unit (URAM) of the International Center for Medical Research of Franceville (CIRMF). This study was conducted from August in December 2015. The enrolled population was consenting adults over 18 years old seen in consultation at CIRMF and having medical tests including uric acid.

### 2.3. Inclusion Criteria

The criteria for selection of the study population were based on previous work carried out in Taiwan [8]. It showed that adult men aged 19 years old and women older than 18 were hyperuricaemic or under treatment. By this observation, only adults over 18 years with a request for uric acid tests have been retained.

For a more rigorous selection of patients, an additional differential examination of streptococci was then used to eliminate from the study all patients presenting with acute articular inflammation of non-gouty origin at the time of sampling. This test called the ASO (the Anti-StreptoLysyne O), has achieved assays serological markers using bsm-antistreptolysin O Kit (MyBiosource, ELISA kits, San Diego, CA, USA). The number of patients selected for this study were 104 patients with uric acid requests corresponding to the selection criteria of the 2972 examinations requested.

### 2.4. Consultation and Data Collection

The survey questionnaire consisted of identifying socio-epidemiological data, clinical and biological parameters, and socio-demographic history. The information sought are mainly those related to environmental and social characteristics of each patient, lifestyle, health and all the features established as risk factors of hyperuricemia and gout.

### 2.5. Assay Method: Serological Assay of ASLO

The collection of 3 to 4 mL of blood was taken by venous access. Serological assay test Anti-StreptoLysyne-O (ASO) was performed by the conventional method on microplates.

The test was performed from the serum, and the tubes were centrifuged at 5000 rpm before carrying out the agglutination test.

Positive and negative controls were also performed. The result of the patient test was compared to the controls, and read semi-quantitatively according to the manufacturer’s recommendations:

After depositing the serum in the 50 μL disk in the presence of the reagent, the patient was declared weakly positive if the appearance of agglutination was observed, and it would have an ASLO level of 200 IU/mL, which would be interpreted as non-recent streptococcal infection.

If, on the other hand, the agglutination was in the 10 μL disk, in this case, the positivity rate would be ≥300 UI/mL and the patient would be considered to have contracted a recent streptococcal infection.

The presence of agglutination in either disc showed that a positive result for streptococcal infection was a cause of joint inflammation in the tested patient.

### 2.6. Biochemical Assay of Serum Uric Acid, Urea and Creatinine

The biochemical tests, including the determination of uricemia, urea and creatinine were made from HITACHI Roche 902 (Roche, diagnostics; F.Hoffmann-La Roche Ltd.; Basel, Switzerland). This automaton uses spectrophotometry as the assay method.

After transferring the samples to the analytical laboratory, they were centrifuged at 5000 rpm for 5 min. In wells, 400 μL of substrate-plasma were introduced, and the wells were deposited in the automaton, which had been previously calibrated and checked. The program of the analysis was scrupulously introduced for each examination requested. The results were then identified and reported in the record of each patient for interpretation.

The normal values of the uric acid were taken according to the standards of the automaton used in the laboratory, i.e., 150 to 360 μmol/L for women and 240 to 420 μmol/L for men.

### 2.7. Definition of Pathological Cases, Evaluation and Interpretation of Parameters

#### 2.7.1. Case of Hyperuricemia

A patient was considered hyperuricaemic when the value of serum uric acid was greater than 360 µmol/L in women and 420 µmol/L in men.

#### 2.7.2. Case of Gout

The definition of gout cases was based on an affirmative answer to the question of whether “a doctor or health professional had diagnosed gout in patients”. The cases of gouty access were, on the other hand, associated with the presence of acute crisis in the patient, and with a result of ASLO negatives.

#### 2.7.3. Case of Renal Insufficiency Type 1 (RI1)

For urea and creatinine, normal value intervals were, respectively, between 1.6 and 8.3 mmol/L and 45 to 140 μmol/L. Urea values and/or higher serum creatinine reveal a critical condition of the kidney or renal dysfunction prodrome, which was defined as a type of kidney disease (RI1).

#### 2.7.4. Evaluation of the Activity of the Tubular Carriers

On the other hand, the biochemical analysis was used to evaluate the metabolic activity of the tubular carriers (due to glomerular filtration) involved in the renal operation, and the glomerular filtration rate (GFR) estimated in milliliters per minute by 1.73 m^2^ was calculated using the formula developed in a previous study [9] on renal disorders for dietary modification. This is stated as the following:

GFR = 175 × concentration of serum creatinine (mg/dL) ^−1.234^ × age ^−0.179^ (× 0.79 for women).

#### 2.7.5. Case of Renal Insufficiency Type 2 and Chronic (Respectively RI2 and RIC)

Two other stages in renal failure have been defined: kidney failure as disease stage 2 (IR2) when GFR < 60 mL/min/1.73 m^2^, and chronic kidney disease (CKD) or type 3 when the GFR < 30 mL/min/173 m^2^ [5]. The creatinine values obtained were expressed in micromoles per liter (μmol/L) and could be converted to milligrams per deciliter (mg/dL) by dividing by 88.46.

#### 2.7.6. Case of Renal Insufficiency Type 2 and Chronic (Respectively IR2 and IRC)

Two other stages in renal failure have been defined: kidney failure as disease stage 2 (IR2) when < 60 GFR mL/min/1.73 m^2^, and chronic kidney disease (CKD) or type 3 when the GFR < 30 mL/min/173 m^2^ [5]. The values of creatinine were obtained expressed in micromoles per liter (µmol/L) and could be converted into milligrams per deciliter (mg/dL) by dividing by 88.46.

#### 2.7.7. Evaluation of Risk Factors for Gout and Hyperuricemia

With or without a family history and sex, as parameters, the GFR has also assessed biochemical genetic factors. Indeed, the tubular urate transporters are encoded by the URAT1 gene (SLC22A12 gene) and GLUT9 (SLC2A9 gene). They provide glomerular filtration in the kidneys, transport and excretion of urate, thus regulating uric acid. In addition, the gene located on chromosome X q26-q27 is implicated in the regulation of serum uric acid in young people. Any change in any of the foregoing genes is a biochemical factor of hyperuricemia and gout [8,10].

Besides kidney function, a correlation with gout and hyperuricemia has been established with other risk factors (or diseases) that were recurrent in patients.

Obesity in patients was defined for body mass index values (BMI) ≥30 kg/m^2^ [10].

#### 2.7.8. Diseases Associated with Gout and Hyperuricemia

High blood pressure (hypertension) was defined for the systolic and diastolic values respectively greater than 140 and 90 mmHg. However, patients with hypertension and known under antihypertensive treatment could directly be included as having values defining a state of hypertension without recheck by medical parameters. Diabetes was assessed following a corresponding diagnostic questions in patients.

Obesity, hypertension (whether or not associated with antihypertensive therapy) or diuretic use, and diabetes are parameters that have been identified and used as biochemical factors that influence the metabolism of the elimination of uric acid. Dysfunction tubular carriers of uric acid was used as biochemical risk factors in evaluating the GFR.

The parameters on feeding behavior (consumption of foods rich in nucleoproteins, alcohol and soda) were assessed on the basis of a questionnaire and taken as biochemical factors influencing the metabolism of purine synthesis by increasing purinosynthesis (hyperactivity of Phosphoribosyl Pyrophosphate synthase).

Genetic factors were evaluated on the basis of the presence of a family history in the gouty patients, and on that of their sex.

### 2.8. Statistical Analysis

The data were entered into an Excel spreadsheet (Microsoft Office 2010, Microsoft, Redmond, WA, USA) and then imported into the software “IBM SPSS Statistics” (version 20, 2011, SPSS Hong-Kong Ltd.; Westland Centre, Hong Kong, China) for statistical analysis. The data were divided into qualitative and quantitative variables. The results of qualitative variables (nominal) are given in numbers (*n*) and percentages (%) relationships have been established with the odds ratio (OR), the relative risk (RR) and the *p*-value (by chi-square test), as appropriate, and confirmed by confidence index (CI 95%) to the ratios by the method of Woolf. The threshold value 0.05 for CI 95% was taken. As for quantitative variables, the mean standards were calculated and compared by *t*-test.

## 3. Results

### 3.1. General Characteristics of the Study Population

From August to December 2015, 2972 examinations request were analyzed at the CIRMF, including 4.2% (*n* = 104) concerning uric acid. Of these 104 patients, 82.7% (*n* = 86) were enrolled and 81.7% (*n* = 85) included. Indeed, after the examination of differential diagnosis (ASO) on the presence of rheumatic inflammation not gouty, one in 86 was positive, which allowed for exclusion from the study. Similarly, 18 (17.3%) other patients were excluded (nine under 18 and nine for refusal of participation).

The 85 patients included in the study consisted of 54% men (*n* = 46) and 46% women (*n* = 39), a sex ratio H/F of 1.2. The average age of the population was 56.55 ± 1.902 years (21–87 years). In addition, preferential sources were 69.4%, 10.6% and 9.4%, respectively, in Franceville (*n* = 59), Sucaf (*n* = 9) and Moanda (*n* = 8), which are among the most populated municipalities in the province of Haut-Ogooué in the southeast.

The distribution of patients according to different socio-professional categories has been established as follows: middle and lower frames (24 patients, 28.24%), executives (29 patients, 34.12%), health workers (10 patients, 11.75%), artisans (7 patients, 8.24%), unemployed and retired (seven patients, 8.24%), farmers (six patients or 7.06%) and students (two patients or 2.35%). grouping these categories gave three classes divided clearly in Table 1.

### 3.2. Prevalence of Hyperuricemia

A total of 30.6% (*n* = 26) of those included had hyperuricemia. Of these included, 23.5% (*n* = 20) were men and 7.1% (*n* = 6) were women. Men were most affected as compared to women (*p*-value = 0.0047). Hyperuricemia reached the majority of the population over 50 years, with an average age of 54.81 ± 1.834 years (OR = 2.6). Furthermore, the average hyperuricemia was highest in Moanda reaching 100%, followed by Sucaf and Franceville, respectively, with 53.1% and 75%.

### 3.3. Gout and Gouty Access Depending on Gender and Age

Among the 85 patients, 15.3% (*n* = 13) had gout, and only men proved to have the disease. Indeed, this prevalence was much higher among those over 50 (*p*-value < 0.0001). In addition, the gout attack, found only in 17.4% (*n* = 8) of men, was present in only 9.4% of the included population. Depending on the age, the gouty crisis rates were significantly similar to those of gout, affecting the population over 50 years (Table 2).

### 3.4. Gout and Gouty Access Depending on Hyperuricemia

Of the 26 hyperuricaemic patients, 46.2% (*n* = 12) were gouty, or 14.1% of the population, and 26.9% (*n* = 7) had acute gout, or 8.2% of the total population. In fact, it appears that hyperuricemia is mainly one of the causes leading to gout (OR = 49.7 and RR = 26.8) and gouty access (OR = 26.2 and RR = 19, 4) *p*-value < 0.0001. Overall, a total of 8.2% (*n* = 7) of patients included were hyperuricaemic and made a gouty access.

Furthermore, among patients enrolled who had a normal uric acid levels, one (1) patient (male) with a serum uric acid concentration of about 220 mmol/L was gouty and exhibited acute crisis.

### 3.5. Range of Biochemical Test Values in the Population

**Figure 1:**
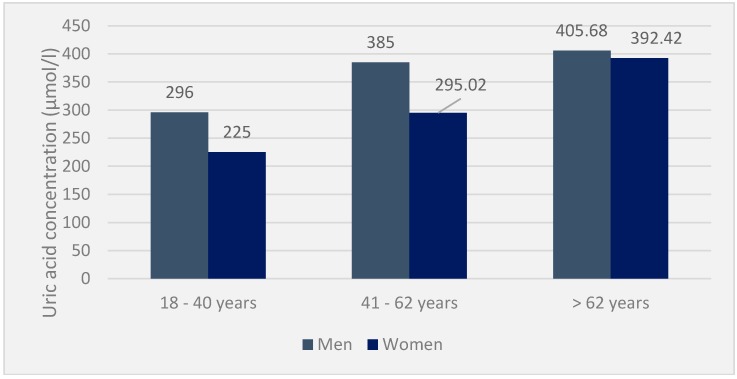
Uricemia in the general population by age and sex (*n* = 85).

In the included population, the mean serum uric acid was 377.9 ± 6 μmol/L, with 403.9 ± 0.8 μmol/L for men versus 351.8 ± 2.2 μmol/L for women. This serum uricemia increases with age and remains, in all cases, higher in men than in women. Mean serum urea and creatinine concentrations were 4.6 ± 0.04 mmol/L and 92.5 ± 5.2 μmol/L, respectively, in the population. In the hyperuricaemic population, the mean value of serum uric acid was 475.13 ± 0.821 μmol/L, i.e., 569.04 ± 1.102 μmol/L in men (495–930 μmol/L) and 381.2 ± 0.540 μmol/L in women (360–525 μmol/L). The average creatinine was 111 ± 3.058 μmol /L, and the urea was 8.4 ± 0.74 mmol/L (Figure 1).

**Figure 2:**
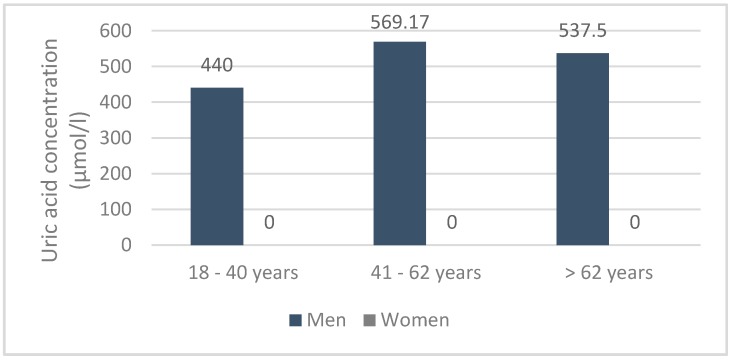
Serum uricemia in the gout population according to age and sex (*n* = 12).

Furthermore, in 12 patients with gouty hyperuricemia, the mean serum uricemia was 425.10 ± 1.484 μmol/L, i.e., 428 ± 0.065 μmol/L (400 and 930 μmol/L) compared to 426.55 ± 2.903 μmol/L. (360 and 430 mmol/L), respectively, in men and women. In contrast, creatinine and urea were around 148.9 ± 1.251 μmol/L and 7.5 ± 0.452 mmol/L, respectively. Finally, serum uricemia was low in patients with acute attacks (425.58 ± 4.562 μmol/L). Depending on the age and sex, serum uric acid was spread in the population, as shown in the following diagram (Figure 2).

### 3.6. Risk Factors and Diseases Associated with Hyperuricemia and Gout

Diets rich in nucleoprotein and alcoholism were recurring in hyperuricaemic patients. They constitute the primary risk factors for hyperuricemia and gout, followed by being overweight. The co-morbidity of cardiovascular diseases associated with the use of diuretics have been determined as the first state of predisposition to elevated uric acid in the blood, in the studied population because 53.8% of hyperuricaemic patients had hypertension. Similarly, diabetes was the disease, after hypertension and renal insufficiency, which was found to be associated with gout in this population (Table 3).

Obesity was found in the included population, with 11.8% (*n* = 10) obese (BMI ≥ 30 kg/m^2^) with an average body mass of 90.43 ± 5.380 kg, for an average BMI of 33.14 ± 1.033 kg/m^2^. Of these patients, seven were hyperuricaemic, including five with hypertension; among them, two with diabetes who had a family history of gout. Most gouty patients were hypertensive. Eleven of the 15 hypertensive patients therefore underwent antihypertensive treatment based on diuretics. A total of 17 patients had prodromal features of renal function disorder. Only a diabetic patient had a family history of gout.

Nearly 90% of patients in the study population have a remarkably easy lifestyle and a diet rich in protein and nucleoelements. The consumption of drinks is very common in this population. Caloric and purine intake of this food could bring about biochemical speciation appearance dietary factors, but they unfortunately could not be assessed. Moreover, this recurrence of the elements rich in purine compounds would make this consumption a risk factor (Table 4).

## 4. Discussion

This study presents the prevalences of hyperuricemia and gout, and shows the different risk factors associated with hyperuricemia and gout in a restricted access adult population in Franceville and its surroundings in the province of Haut-Ogooué in the southeast. Biochemical implications have been established. Moreover, the enumeration of hyperuricaemic cases has been made possible by the spectrophotometric assay (Roche integrated into HITACHI 902) of uric acid in the plasma of each subject. Cases of Acute Articular Rheumatism (AAR) of non-gouty origin were determined by the agglutination method implemented in the ASLO kit. Without any ambiguity and no bias in the results, HITACHI option Roche 902, just like the differential test of RAA (ASLO) could be used for the assay of a rather high and representative sampling. Indeed, the use of the HITACHI Roche automaton in the determination of uric acid in blood and urine has already been applied [11]. Women and men is approximately equal to 46% of women and 54% of men. This will reduce the bias in comparisons between sexes on different aspects studied. However, methodological shortcomings must be taken into account in the interpretation of the results obtained, especially in the determination of gout and gouty crisis. The assay of C-reactive protein (CRP) and determining the speed of sedimentation (SS) would have been required in the characterization of inflammatory reactions, for the deduction of a gout. In addition, the estimate rates of morbidity of the hyperuricemia and gout remains to appreciate if other later works include a representative sampling.

Studies have shown that hyperuricemia is unevenly distributed by sex and age. The prevalence of hyperuricemia is much higher in men, with 23.5% compared to 7.06% in women (OR = 4.28). In addition, gouty access is only found in men over 49 years of age, although weakly representative provincially. The ubiquity character of gout and hyperuricemia are demonstrated, and these results have showed that gout would be quite important in the general population of this province, and also suggested that men would be more exposed to hyperuricemia and gout as women. The results of this study are in concordance with those published in 1992 where the prevalence of hyperuricemia was a male tendency [7]. Other studies conducted outside the West have attested to the high character of uric acid in Samoa men, with 36.4% of men against 14.7% of women [12]. In Togo, the prevalence of gout has shown that only one woman out of 159 cases was affected [13]. Moreover, the latest data published in 2012 in the United States showing that the relative physiological impact of having gout or a certain rate of hyperuricemia is higher in women than men [5], however, are contradictory to results that have been achieved. A case-control study would be ideal to establishing a clear limit of physiological influences on serum uricemia, and it is increasing in men and women in a larger population of Haut-Ogooué and other provinces of Gabon.

In general, hyperuricaemic patients would be gouty and/or would have gouty access. Indeed, of the 26 hyperuricaemic counted, 12 were gouty and seven had acute crisis. Hyperuricemia is thus the main factor of gout (OR = 49.7). In contrast, a single case, with normal uricemia, was gouty and acute gouty access in this case was demonstrated in 2010 [13]. Further studies could allow the distribution of chronic arthritis and rheumatism, by age and gender and set a specific limit (if possible) of uricemia in gout affected patients.

In hyperuricaemic patients, the values of serum uricemia are very high, reaching 930 μmol/L in men over 45 years of age. These higher concentrations in men partly explain their predisposition to gout. The evolution of serum uricemia in the population is increasing for both men and women but remains higher among men regardless of age. There exist specific factors peculiar to men who are the cause of these high values, or, conversely, a set of mechanisms present in women would regulate the metabolism of uric acid. These factors could be genetic and/or hormonal in nature. It is only at menopause that uric acid levels are approaching in men and women [14].

Authors have demonstrated in 2002 that chronic juvenile arthritis (CJA) is very rare, especially in sub-Saharan Africa, which explains a low concentration of uric acid in young people [2]. These prevalences are unchanged in this study. However, the 25 cases of gout in the Congo show that young people are not necessarily free from pathological uric acid levels [8]. The relative explanation, corroborating with the fact that uric acid is low and cases of gout are negligible in the juvenile age group (under 20 years), would be the right activity of purine metabolism actors and low speed of constitution of monosodium crystals [13].

Alcoholism (OR = 13), obesity or being overweight (OR = 6.9), family history (OR = 6.8), kidney failure (OR = 4.4), and hypertension (OR = 1.7) were risk factors commonly associated with hyperuricemia included in the population. In addition, hyperuricemia (OR = 49.7) and the foregoing factors (OR = 1) would be the risk factors of gout in adult patients in the population of the southeast in Gabon. These risk factors were found respectively in 30.6% (*n* = 26), 97.6% (*n* = 83), 11.8% (*n* = 10), 8.2% (*n* = 7), 13.1% (*n* = 23) and 44.7% (*n* = 38) of patients. At least one of these factors was present in 24 of 26 hyperuricemic patients (95.6%). The specific factors are those conventionally reported in the literature. The methodology adopted did not identify potential risk factors specific to residents of the province. Determination of attributable risk could have classified these factors according to their real involvement in the pathologies studied. However, there was little rigorous data on obesity, high blood pressure, alcoholism, and especially diabetes and the family history of our population. It was therefore not possible to quantify attributable risk. The association of gout with alcoholism, obesity, hypertension, renal failure and family history in patients is traditional and consistent with previous studies in Togo [12].

The results were also consistent with the observations made in the Congo [15], where alcoholism, obesity and high blood pressure were observed in, respectively, 53.3% (*n* = 32), 46.7% (*n* = 28) and 30% (*n* = 18) patients. Observation of dietary factors is correlated with trends in the Europeanization of the diets of African people especially in Gabon. In sub-Saharan Africa, obesity coexists with malnutrition and mainly affects the urban population [12].

The results of the urea and creatinine examinations have established the biochemical causal relationships of the factors predisposing to a state of hyperuricemia and gouty access. This does not represent an exhaustive list, since these interpretations were based on values derived from parameters such as GFR (Globular Filtration Rate), the qualitative food intake, by correlation with the activity of the metabolites involved in the synthesis and the elimination of uric acid.

Alcohol and diets rich in purine compounds involve an increase in the synthesis of lactate, acetyl-CoA and AMP, and that could cause an overproduction of uric acid [16]. They would also be responsible for a decrease in biochemical activity tubular carriers and elimination of uric acid [17]. It could be the same for the renal failure (21.68%) of the population. Taking diuretics may also be responsible for increasing uric acid, more than these interacting with metabolites regulating filtration and uric acid removal [8,9].

However, some limitations of the study that were conducted are to be underlined. The number of consultations in medical centers in all the provinces of Gabon concerning the diagnosis of gout is a limiting factor because it is extremely weak. Thus, many patients will never receive appropriate care when the disease is discovered very late. This will have the effect of increasing the number of patients to acute access.

In this study, the total study population allowed us to establish the prevalence and identify the risk factors associated with gout. With a population of ≥500 individuals, we will be able to confirm certain parameters, in particular to establish the difference between men and women in the face of the disease and to determine the critical age between the two sexes.

It would also be desirable to assay the PRPP synthase to quantitatively establish the metabolic and biochemical profile of each patient.

Therefore, the low representation of social groups most affected by the disease probably introduced bias into the results.

## 5. Conclusions

This study has confirmed the ubiquitous nature of gouty arthropathies, given the remarkable rates of hyperuricemia and gouty access highlighted in the included population. In addition, classic risk factors for gout and hyperuricemia have been identified in this population, including: alcoholism, obesity, protein overeating, and family history. Added to this, diseases associated with gout have been revealed to be hypertension, diabetes and renal insufficiency. Risk factors associated with the disease commonly found in the vast majority of the population constitute a handicap for this society. These results revealed a masculine trend of gouty attacks, gout and hyperuricemia, and involve a lot more older people. Given the increasing prevalence of hyperuricemia and gout from its discovery to the present day, and complications that can be entrained by the co-morbidity of hyperuricemia and gout associated with other diseases, these conditions must be recognized and taken into account in the long-term management and overall health of people with gout (respectively, gouty access) and hyperuricemia. Therefore, further studies are needed to confirm these facts, firstly to quantify the impact of conventionally known factors and also detect possible risk factors for Gabonese people.

## Figures and Tables

**Table 1:** General characteristics of the study population.

Characteristics		Men	Women	Total	*p*-Value
Sex		46	39	85	<0.0047
Age (years)		43.33 ± 1.08	69.77 ± 2.71	56.55 ± 1.90	0.0089
Body Mass (Kg)		74.04 ± 0.20	66.04 ± 8.57	76.04 ± 9.08	0.0261
BMI (Kg/m^2^)		27.95 ± 2.01	26.17 ± 1.09	27.57 ± 3.5	0.0435
AHT, *n* (%)		17 (37.0)	22 (56.2)	39 (45.8)	0.0411
Uricemia (µmol/L)		403.9 ± 9.8	351.8 ± 2.2	377.9 ± 6	<0.0001
Creatine (µmol/L)		108.04 ± 1.1	77.04 ± 9.1	92.5 ± 5.1	<0.0001
Urea (mmol/L)		4.2 ± 0.7	5.0 ± 0.1	4.6 ± 0.4	<0.0001
Diabetes, *n* (%)		5 (10.9)	8 (20.5)	13 (15.3)	0.0499
Hyperuricemia *n* (%)	<50 years	8 (17.4)	0 (0.0)	8 (9.4)	0.053
	≥50 years	18 (39.1)	6 (15.4)	22 (25.9)	<0.0001
Gouty *n* = 13 (%)	<50 years	3 (6.5)	0 (0.0)	3 (3.5)	-
	≥50 years	10 (21.7)	0 (0.0)	10 (14.1)	<0.0001
Gty acc. *n* = 8 (%)	<50 years	1 (2.2)	0 (0.0)	1 (9.4)	
	≥50 years	7 (17.4)	0 (0.0)	7 (8.2)	<0.0001
Gout Fam.Ant. *n* (%)		7 (15.2)	2 (5.1)	9 (10.6)	0.0059
Food. Drn. *n* (%)		46 (100)	39 (100)	85 (100)	0.0494
Alcohol and soda, *n* (%)		35 (76.1)	25 (64.1)	60 (70.6)	<00001
Source, *n* (%)	Franceville	32 (69.6)	27 (69.2)	59 (69.4)	
	Other	14 (30.4)	12 (30.8)	26 (30.6)	
Social cat. *n* (%)	Upper cat.	35 (76.1)	9 (23.1)	44 (51.8)	0.0024
	Middle cat.	4 (8.7)	13 (33.33)	17 (20)	0.0015
	Low cat.	7 (15.2)	17 (43.6)	24 (61.5)	

BMI: Body Mass Index; AHT: Arterial hypertension: Gty acc.: Gouty access; Gout Fam. Ant.: Gout Family Antecedent; Food. Drn.: Diet rich in nucleoproteins; Social cat.: Social category.

**Table 2:** Prevalence of hyperuricemia, gout and gouty access in the study population by sex and age.

		Total (N = 85)	Hyperuricemia (N = 26)	Gouty (N = 13)	Gouty Access (N = 8)
		*n* %	*n* % CI95%	*n* % CI95%	*n* % CI95%
Sex	Men	46, 54.1	20, 43.5	13, 28.3	8, 17.4
	Women	39, 45.9	6, 5.3	0, 0.0	0, 0.0
Age (Years)	<50	23, 27.1	4, 17.4, 0.08–11	3, 13, 0.01–3.4	1, 4.3, 0.9–7.6
	≥50	62, 72.9	22, 35.5, **0.4–0.9**	10, 16.1, **1.6–18**	7, 11.3, **7–9.13**

N = Total number and *n* = number of men and number of women; 95% CI = confidence interval; bold indicates significant statistic values with (OR > 1 and *p*-value < 0.05).

**Table 3:** Risk factors and diseases associated with hyperuricemia, gout and/or gouty access in the included population (*n* = 85).

Parameters		Total (N = 85)		Hyperuricemia (N = 26)			Gout (N = 13)			Gty. Acc. (N = 8)		
		*n*	%	*n*	%	OR	*n*	%	OR	*n*	%	OR
Food. Drn.		79	92.9	26	100	00	13	100	00	8	100	00
Hyperuricemia		26	30.6	-	-	-	12	92.3	49.7	7	85.7	26.2
Alcohol and soda		83	97.6	22	84.6	13	9	69.2	8.8	7	85.5	1.9
Obesity (BMI ≥ 30 kg/m^2^)		10	11.8	7	26.9	6.9	3	23.1	2.8	1	12.5	1
AHT and diuretics		38	44.7	14	53.8	1.7	5	38.5	0.7	4	50.0	0.8
Renal Insufficiency (*n* = 23)	RI1	19	22.4	7	29.9	1.4	6	46.2	3.9	4	50.0	0.2
	RI2	13	15.3	9	34.6	7.3	6	46.2	8.0	3	0.4	0.3
	CKD	1	1.2	1	3.8	00	1	7.7	00	1	12.5	00
Fam. Ant.		7	8.2	5	19.2	6.8	1	7.7	1.8	1	12.5	1.7

BMI: Body Mass Index; AHT: Arterial hypertension: Gty Acc.: Gouty access; CKD: chronic kidney disease; Fam. Ant: Family Antecedent; Food. Drn.: Diet rich in nucleoproteins; RI1 (creatinine ≥ 140 μmol/L and urea ≥ 8.3 mmol/L); RI2 (GFR < 60 mL/min/173 m^2^).

**Table 4:** Hyperuricemia, gout and gouty access by social category.

Social Category	Hyperuricemia	Gout	Gouty Access
Upper category *	16 (18.82%)	6 (7.06%)	4 (4.70%)
Middle category **	3 (3.53%)	3 (3.53%)	3 (1.18%)
Low category ***	7 (8.24%)	4 (4.70%)	3 (1.18%)

* Senior managers/health staff; ** middle and lower management/students/artisans/farmers; *** unemployed/retired.
